# Clinical significance of obstructive sleep apnea in patients with acute coronary syndrome in relation to diabetes status

**DOI:** 10.1136/bmjdrc-2019-000737

**Published:** 2019-12-18

**Authors:** Xiao Wang, Jingyao Fan, Yunhui Du, Changsheng Ma, Xinliang Ma, Shaoping Nie, Yongxiang Wei

**Affiliations:** 1 Emergency and Critical Care Center, Beijing Anzhen Hospital, Capital Medical University, Beijing, China; 2 Beijing Key Laboratory of Upper Airway Dysfunction-Related Cardiovascular Diseases, Beijing Institute of Heart, Lung and Blood Vessel Diseases, Beijing, China; 3 Department of Cardiology, Beijing Anzhen Hospital, Capital Medical University, Beijing, China; 4 Department of Emergency Medicine, Thomas Jefferson University, Philadelphia, Pennsylvania, USA; 5 Department of Otolaryngology Head & Neck Surgery, Beijing Anzhen Hospital, Capital Medical University, Beijing, China

**Keywords:** acute coronary syndrome, type 2 diabetes, obstructive sleep apnea syndrome, outcomes

## Abstract

**Objective:**

The prognostic significance of obstructive sleep apnea (OSA) in patients with acute coronary syndrome (ACS) according to diabetes mellitus (DM) status remains unclear. We aimed to elucidate the association of OSA with subsequent cardiovascular events in patients with ACS with or without DM.

**Research design and methods:**

In this prospective cohort study, consecutive eligible patients with ACS underwent cardiorespiratory polygraphy between June 2015 and May 2017. OSA was defined as an Apnea Hypopnea Index ≥15 events/hour. The primary end point was major adverse cardiovascular and cerebrovascular events (MACCEs), including cardiovascular death, myocardial infarction, stroke, ischemia-driven revascularization, or hospitalization for unstable angina or heart failure.

**Results:**

Among 804 patients, 248 (30.8%) had DM and 403 (50.1%) had OSA. OSA was associated with 2.5 times the risk of 1 year MACCE in patients with DM (22.3% vs 7.1% in the non-OSA group; adjusted HR (HR)=2.49, 95% CI 1.16 to 5.35, p=0.019), but not in patients without DM (8.5% vs 7.7% in the non-OSA group, adjusted HR=0.94, 95% CI 0.51 to 1.75, p=0.85). Patients with DM without OSA had a similar 1 year MACCE rate as patients without DM. The increased risk of events was predominately isolated to patients with OSA with baseline glucose or hemoglobin A1c levels above the median. Combined OSA and longer hypoxia duration (time with arterial oxygen saturation <90%>22 min) further increased the MACCE rate to 31.0% in patients with DM.

**Conclusions:**

OSA was associated with increased risk of 1 year MACCE following ACS in patients with DM, but not in non-DM patients. Further trials exploring the efficacy of OSA treatment in high-risk patients with ACS and DM are warranted.

**Significance of this study**

**What is already known about this subject?**

- Prior reports indicated that obstructive sleep apnea (OSA) may be associated with increased risk of recurrent cardiovascular events after acute coronary syndrome (ACS).
- Whether the prognostic significance of OSA in patients with ACS may be modified by diabetes mellitus (DM) status remains unclear.

**What are the new findings?**

- In this prospective cohort study involving 804 patients with ACS, OSA was associated with 2.5 times the risk of major adverse cardiovascular and cerebrovascular events (MACCEs) in patients with DM, but not in patients without DM. patients with DM without OSA had a similar 1 year MACCE rate as patients without DM.
- The increased risk of adverse events was predominately isolated to patients with OSA with poor glucose control.

**How might these results change the focus of research or clinical practice?**

- The present study showed that OSA was only associated with increased risk of adverse events following ACS in patients with DM, therefore representing a high-risk subset most likely to respond to the intervention.
- Future trials evaluating the effects of OSA treatment in patients with DM and ACS are needed.

## Introduction

Type 2 diabetes mellitus (DM) is associated with extensive coronary atherosclerosis^1^ and accelerated plaque progression.^2–4^ Compared with non-diabetics, patients with DM are known to have a higher risk of recurrent cardiovascular events after acute coronary syndromes (ACS).^5–7^ Obstructive sleep apnea (OSA) is an increasingly recognized chronic disorder in patients with ACS, with reported incidence of 36% to 63% across various ethnicities.^8^ Recent evidence indicates OSA initiates and exacerbates coronary atherosclerosis,^9–11^ and predicts adverse cardiovascular outcomes post-ACS in the long run.^12–16^ Also, studies have shown the potential interaction between OSA and DM in glycemic control and the development of chronic diabetic complications.^17–20^ Whether the prognostic significance of OSA in patients with ACS would vary according to DM status has not been characterized. Given the shared mechanisms by OSA and DM in promoting atherosclerosis, we hypothesized that concomitant OSA and DM might exert a synergistic deleterious effect increasing future cardiovascular risk. Therefore, we performed a large prospective cohort study and elucidated the association of OSA with subsequent cardiovascular events in patients with ACS in relation to diabetes status.

## Research design and methods

### Study design and participants

This was a prospective cohort study to evaluate the impact of OSA on cardiovascular outcomes in patients presenting with ACS stratified by diabetes. The study design has been described previously.^21^ In brief, from June 2015 to May 2017, a total of 899 patients aged 18 years to 85 years and admitted for ACS were consecutively enrolled and received overnight sleep study in the Emergency and Critical Care Center of Beijing Anzhen Hospital, Capital Medical University. Exclusions included cardiogenic shock (n=5), cardiac arrest (n=6), malignancy (n=8), and failed sleep study (patients without adequate and satisfactory signal recording, n=13). This left 867 patients with a successful sleep study. Next, patients with predominantly central sleep apnea (≥50% central events or a central Apnea Hypopnea Index (AHI) ≥10/hours, n=20) and those receiving regular continuous positive airway pressure (CPAP) therapy (>3 months, n=10) after discharge were excluded. Thirty-three patients were lost to follow-up and were excluded from the analysis. The final cohort included 804 patients. This study conformed to the STrengthening the Reporting of OBservational studies in Epidemiology guidelines, and was conducted in accordance with the amended Declaration of Helsinki. All patients provided written informed consent.

All patients received standard care during hospitalization according to current guidelines.^22 23^ Type 2 DM was identified by patient history, supported by treatment details. Glucose and hemoglobin A1c (HbA1c) levels were obtained from non-fasting venous blood samples at admission. We described clinical, demographic, angiographic and procedural characteristics for each group according to DM status.

### Overnight sleep study

The diagnosis of OSA was assessed by an overnight portable cardiorespiratory polygraphy (ApneaLink Air, Resmed, Australia) after clinical stabilization during hospitalization (median 2 days (1 to 3 days) after admission). Sleep studies were scored according to the American Academy of Sleep Medicine 2007 guidelines. Sleep studies of <3 hours of satisfactory signal recording were considered invalid and excluded from analysis. All studies were scored manually twice (by XW and JF), both of whom were blinded to the demographic and clinical characteristics. Further analysis was performed in cases of discrepancy by a senior consultant in sleep medicine (YW). Nasal airflow, thoracoabdominal movements, pulse oximetry, and snoring episodes were recorded. An apnea was defined as total cessation of airﬂow for ≥10 s (obstructive if thoracoabdominal movement was present, and central if thoracoabdominal movement was absent). A hypopnea was defined as an airflow reduction of 30% for ≥10 s and was associated with a drop in arterial oxygen saturation (SaO_2_) >4%. The total AHI was defined as the number of apneas and hypopneas per hour of total recording time. OSA was defined as AHI ≥15 events/hour. Patients with AHI <15 events/hour were considered as the non-OSA group. Patients with OSA (AHI ≥15), particularly those with excessive daytime sleepiness, were referred to the sleep center for further evaluation.

### End points

The primary end point was major adverse cardiovascular and cerebrovascular event (MACCE), defined as a composite of cardiovascular death, myocardial infarction, stroke, ischemia-driven revascularization, or hospitalization for unstable angina or heart failure. Secondary end points included the components of primary end point, all-cause death, repeat revascularization, and a composite of all events. All end points were deﬁned according to the proposed deﬁnitions by the Standardized Data Collection for Cardiovascular Trials Initiative.^24^ Patients were followed up from the time of the sleep study and at 1 month, 3 months, 6 months, 1 year, and every 6 months thereafter. Clinical events were collected via clinic visit, medical records, or telephone calls and led by an investigator (SN) who was blinded to the patients’ sleep results. All clinical events were independently adjudicated by the clinical event committee blinded to the OSA and DM status. The committee also reviewed the source documents and established the necessity for hospital admission and/or revascularization.

### Statistical analyses

We reported the impact of OSA on MACCE and other cardiovascular events by DM status, the median of serum glucose (5.9 mmol/L), and the median of HbA1c (6.0%). Kaplan-Meier curves were plotted in OSA and non-OSA groups stratified by DM status, dichotomized serum glucose and HbA1c, and compared by log-rank test. The relationship between OSA and the end points by DM status were analyzed by Cox proportional hazard models. Baseline variables that were considered clinically relevant or that showed a univariate relationship with outcome were entered into multivariable Cox regression models. Variables for inclusion were carefully chosen, given the number of events available, to ensure parsimony of the final models. If a patient experienced more than one event, only the first event was included in the analysis. Adjusted HR with 95% CI was calculated.

Receiver operating curve (ROC) analyses were performed to identify parameters of OSA to predict MACCE in patients with DM. The optimal cut-off value was estimated by area under the curve (AUC) via Youden’s Index (the maximum value of (sensitivity+specificity − 1)). We calculated event rates based on single or combined OSA characteristics.

Continuous variables were presented as mean±SD or median (first and third quartiles), and were compared by Student’s *t*-test or Mann-Whitney U test. Categorical variables were shown as the number (percentage), and were compared using χ^2^ statistics or Fisher’s exact test, when appropriate. All analyses were conducted with SPSS V.22.0 (IBM SPSS, Armonk, New York, USA). A two-sided *p* value<0.05 was considered statistically significant.

## Results

### Patients

A total of 804 patients (82.6% male; 57.5±10.2 years) was included in the final analysis. Of these, 248 (30.8%) had DM and 403 (50.1%) had OSA. Patients with DM were older, more likely to be female, and had a higher incidence of hypertension and hyperlipidemia, but were less likely to be current smokers. The prevalence of OSA was similar in both DM and non-DM groups. Sleep study results were comparable between DM and non-DM patients (online supplementary eTable 1).

10.1136/bmjdrc-2019-000737.supp1 Supplementary data

The baseline characteristics of OSA and non-OSA groups according to DM status are presented in table 1 and online supplementary eTable 2. Patients with OSA exhibited higher body mass index and waist-to-hip ratio in both subgroups. Baseline glucose and HbA1c levels tended to be higher in patients with OSA, irrespective of DM status. Multivessel disease was more frequent in the OSA group compared with the non-OSA group, with numerically more percutaneous coronary intervention (PCI) procedures in the OSA group. Other information was generally well matched between OSA and non-OSA patients in the DM and non-DM subgroups.

**Table 1:** Baseline patient characteristics in the OSA versus non-OSA groups according to diabetes status

Variables	All (n=804)	Diabetes (n=248)			No diabetes (n=556)		
		OSA (n=121)	Non-OSA (n=127)	P value	OSA (n=282)	Non-OSA (n=274)	P value
Demographics							
Age, years	57.5±10.2	59.4±9.6	58.7±9.7	0.54	56.9±10.4	56.5±10.3	0.64
Male	664 (82.6)	91 (75.2)	97 (76.4)	0.83	251 (89.0)	225 (82.1)	0.02
BMI, kg/m^2^	26.7±3.6	27.4±3.0	26.1±3.1	0.001	27.7±3.7	25.6±3.5	<0.001
Waist-to-hip ratio	0.98 (0.96 to 1.02)	0.99 (0.96 to 1.03)	0.98 (0.95 to 1.01)	0.009	0.99 (0.96 to 1.02)	0.98 (0.93 to 1.01)	0.001
Neck circumference, cm	40 (38 to 42)	41 (39 to 43)	40 (38 to 42)	0.003	41 (39 to 43)	39 (37 to 41)	<0.001
Medical history							
Hypertension	530 (65.9)	92 (76.0)	95 (74.8)	0.82	183 (64.9)	160 (58.4)	0.12
Hyperlipidemia	210 (26.1)	43 (35.5)	41 (32.3)	0.59	64 (22.7)	62 (22.6)	0.99
Prior stroke	76 (9.5)	20 (16.5)	17 (13.4)	0.49	21 (7.4)	18 (6.6)	0.69
Prior myocardial infarction	118 (14.7)	20 (16.5)	22 (17.3)	0.87	41 (14.5)	35 (12.8)	0.55
Prior PCI	141 (17.5)	27 (22.3)	24 (18.9)	0.51	56 (19.9)	34 (12.4)	0.02
Prior CABG	11 (1.4)	6 (5.0)	2 (1.6)	0.16	3 (1.1)	0 (0.0)	0.25
Smoking				0.44			0.79
No	288 (35.8)	56 (46.3)	49 (38.6)		89 (31.6)	94 (34.3)	
Current	406 (50.5)	49 (40.5)	61 (48.0)		153 (54.3)	143 (52.2)	
Previous	110 (13.7)	16 (13.2)	17 (13.4)		40 (14.2)	37 (13.5)	
Baseline tests							
Glucose, mmol/L	5.9 (5.3 to 7.3)	8.5 (6.8 to 11.0)	7.4 (6.2 to 9.3)	0.008	5.7 (5.2 to 6.3)	5.5 (5.1 to 6.1)	0.07
Hemoglobin A1c, %	6.0 (5.6 to 6.9)	7.8 (6.7 to 9.2)	7.2 (6.4 to 8.4)	0.006	5.8 (5.5 to 6.1)	5.7 (5.4 to 6.1)	0.02
Hs-CRP, mg/L	2.3 (0.8 to 8.6)	2.7 (1.0 to 8.9)	1.7 (0.7 to 4.8)	0.03	3.0 (1.1 to 11.6)	1.9 (0.6 to 6.8)	0.001
LVEF, %	60 (55 to 65)	60 (55 to 65)	63 (57 to 66)	0.14	60 (56 to 65)	60 (55 to 65)	0.92
Diagnosis				0.20			0.31
Unstable angina	347 (43.2)	59 (48.8)	66 (52.0)		120 (42.6)	102 (37.2)	
NSTEMI	203 (25.2)	25 (20.7)	34 (26.8)		66 (23.4)	78 (28.5)	
STEMI	254 (31.6)	37 (30.6)	27 (21.3)		96 (34.0)	94 (34.3)	
Procedures							
Coronary angiography	786 (97.8)	119 (98.3)	123 (96.9)	0.68	278 (98.6)	266 (97.1)	0.22
Multivessel disease	61.2 (481/786)	88/119 (73.9)	69/123 (56.1)	0.004	177/278 (63.7)	147/266 (55.3)	0.046
PCI	490 (60.9)	74 (61.2)	66 (52.0)	0.15	188 (66.7)	163 (59.5)	0.08
CABG	80 (10.0)	14 (11.6)	17 (13.4)	0.67	23 (8.2)	26 (9.5)	0.58
Medications on discharge							
Aspirin	754 (93.8)	112 (92.6)	116 (91.3)	0.72	269 (95.4)	257 (93.8)	0.41
Thienopyridine	720 (89.6)	107 (88.4)	111 (87.4)	0.80	262 (92.9)	240 (87.6)	0.03
β-blockers	611 (76.0)	91 (75.2)	97 (76.4)	0.83	222 (78.7)	201 (73.4)	0.14
ACEIs/ARBs	564 (70.1)	83 (68.6)	96 (75.6)	0.22	208 (73.8)	177 (64.6)	0.02
Statins	762 (94.8)	114 (94.2)	116 (91.3)	0.38	271 (96.1)	261 (95.3)	0.63

Data are presented as mean±SD, median (IQR), n (%), or n/N (%).

ACEI, ACE inhibitor; ARB, angiotensin receptor blocker; BMI, body mass index; CABG, coronary artery bypass grafting; Hs-CRP, high-sensitivity C reactive protein; LVEF, left ventricular ejection fraction; NSTEMI, non-ST segment elevation myocardial infarction; OSA, obstructive sleep apnea; PCI, percutaneous coronary intervention; STEMI, ST segment elevation myocardial infarction.

### Outcomes of OSA versus non-OSA patients in relation to diabetes status and glucose control

**Figure 1:**
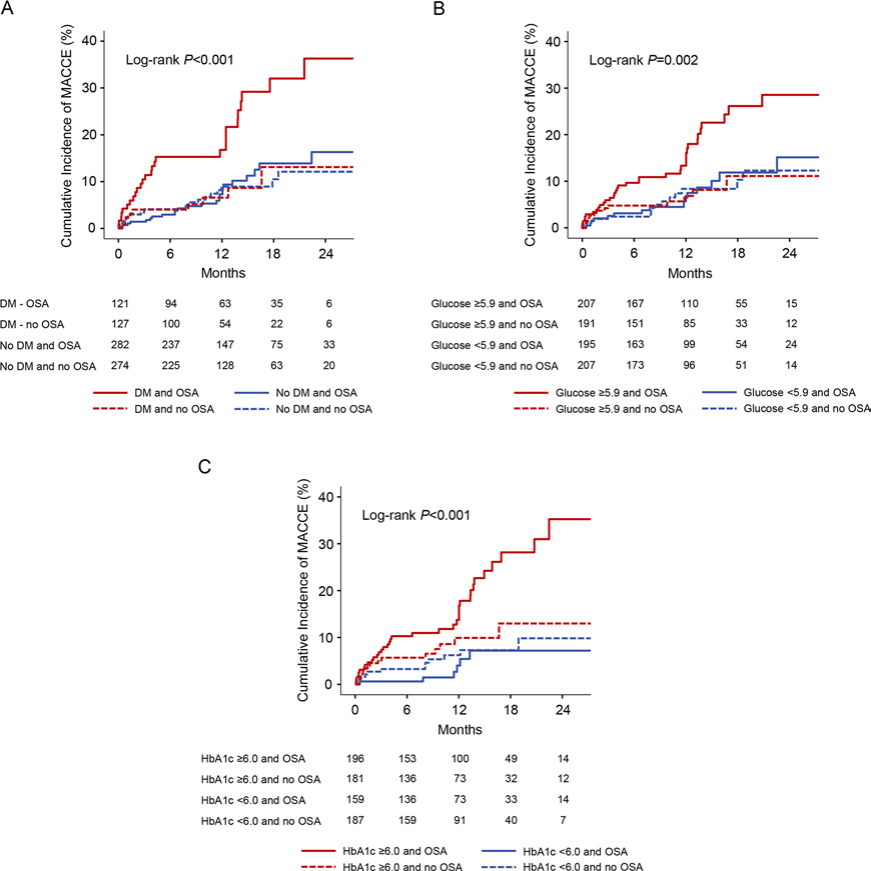
Kaplan-Meier curves for MACCE in OSA versus non-OSA groups according to diabetes status and glucose control. The cumulative incidences of MACCE are shown in OSA and non-OSA groups, stratified on the basis of diabetes or no diabetes (A), glucose level above or below the median of 5.9 mmol/L (B), and HbA1c above or below the median of 6.0% (C). DM, diabetes mellitus; HbA1c, hemoglobin A1c; MACCE, major adverse cardiovascular and cerebrovascular event; OSA, obstructive sleep apnea.

**Figure 2:**
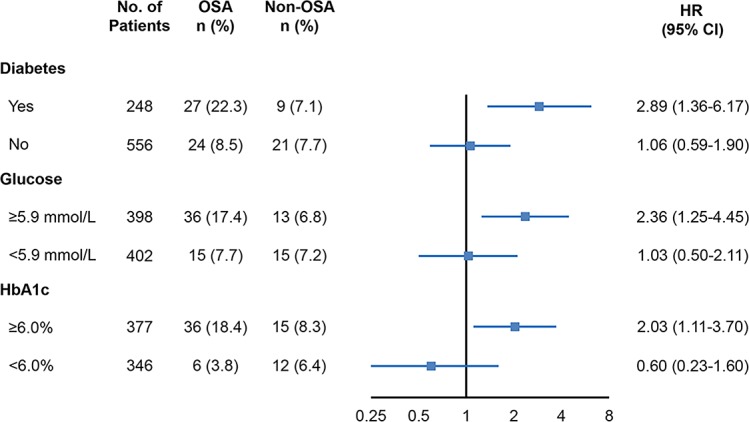
Outcomes of MACCE in OSA vs non-OSA groups according to diabetes status and glucose control.Shown are the crude incidences of MACCE, unadjusted HR, and 95% CI in patients with OSA and non-OSA patients in each subgroup. HbA1c, hemoglobin A1c; MACCE, major adverse cardiovascular and cerebrovascular event; OSA, obstructive sleep apnea.

Among patients with DM, the presence of OSA was strongly associated with a higher rate of MACCE compared with patients without OSA (22.3% vs 7.1%; HR=2.89, 95% CI 1.36 to 6.17, p=0.006). In contrast, the incidence of MACCE was similar between OSA and non-OSA groups in patients without DM (8.5% vs 7.7%; HR=1.06, 95% CI 0.59 to 1.90, p=0.85; table 2, figures 1A and 2). A similar finding was observed in multivariable Cox regression analysis (table 2). Patients with DM without OSA had a similar 1 year rate of MACCE as all patients without DM (7.1% vs 8.1%; HR=0.97, 95% CI 0.47 to 1.98, p=0.93). There was also no significant difference in the incidence of MACCE in DM and non-DM patients without OSA (7.1% vs 7.7%; HR=0.99, 95% CI 0.45 to 2.15, p=0.97). Conversely, in patients with OSA, the 1 year MACCE rate was greater in patients with DM compared with non-DM patients (22.3% vs 8.5%; HR=2.70, 95% CI 1.56 to 4.67, p<0.001). There was a trend towards increased risk of hospitalization for unstable angina in OSA versus non-OSA groups in patients with DM (table 2). Other crude number of events are listed in online supplementary eTable 3.

**Table 2:** Clinical outcomes in OSA versus non-OSA groups according to diabetes status

Variables	Diabetes				No diabetes			
	Unadjusted HR (95% CI)	P value	Adjusted HR* (95% CI)	P value	Unadjusted HR (95% CI)	P value	Adjusted HR* (95% CI)	P value
MACCE	2.89 (1.36 to 6.17)	0.006	2.49 (1.16 to 5.35)	0.02	1.06 (0.59 to 1.90)	0.85	0.94 (0.51 to 1.75)	0.85
Cardiovascular death†	5.11 (0.60 to 43.77)	0.14	–	–	–	0.25	–	–
Myocardial infarction†	2.75 (0.28 to 26.72)	0.38	–	–	0.16 (0.02 to 1.31)	0.09	–	–
Stroke†	3.98 (0.44 to 35.73)	0.22	–	–	0.95 (0.13 to 6.74)	0.96	–	–
Ischemia-driven revascularization	1.52 (0.50 to 4.66)	0.47	1.50 (0.48 to 4.68)	0.49	1.64 (0.55 to 4.89)	0.38	1.35 (0.42 to 4.30)	0.62
Hospitalization for unstable angina	2.49 (0.97 to 6.38)	0.06	2.41 (0.93 to 6.26)	0.07	1.56 (0.71 to 3.40)	0.27	1.45 (0.64 to 3.33)	0.38
Hospitalization for heart failure†	–	0.61	–	–	0.64 (0.11 to 3.85)	0.63	–	–
All repeat revascularization	1.81 (0.67 to 4.90)	0.25	1.86 (0.68 to 5.14)	0.23	1.13 (0.56 to 2.28)	0.74	1.08 (0.51 to 2.28)	0.85
Composite of all events	2.89 (1.40 to 5.93)	0.004	2.52 (1.21 to 5.22)	0.01	1.02 (0.62 to 1.67)	0.94	0.95 (0.56 to 1.62)	0.86

*Model adjusted for age, sex, body mass index, hypertension, and diabetes mellitus, clinical presentation (acute myocardial infarction vs unstable angina).

†Univariate and/or multivariate Cox regression was not done due to no or few number of events.

MACCE, major adverse cardiovascular and cerebrovascular event; OSA, obstructive sleep apnea.

For patients with glucose levels above the median of 5.9 mmol/L, the presence of OSA was associated with a higher incidence of MACCE compared with patients without OSA (17.4% vs 6.8%; HR=2.36, 95% CI 1.25 to 4.45, p=0.008; figures 1B and 2). Likewise, an increased risk of MACCE was observed in the OSA group among patients with HbA1c levels above the median of 6.0% (18.4% vs 8.3%; HR=2.03, 95% CI 1.11 to 3.70, p=0.022; figures 1C and 2). In contrast, there were no significant differences in MACCE rates among patients with glucose or HbA1c lower than median levels (figure 2).

### OSA-related characteristics to predict MACCE in patients with DM

**Figure 3:**
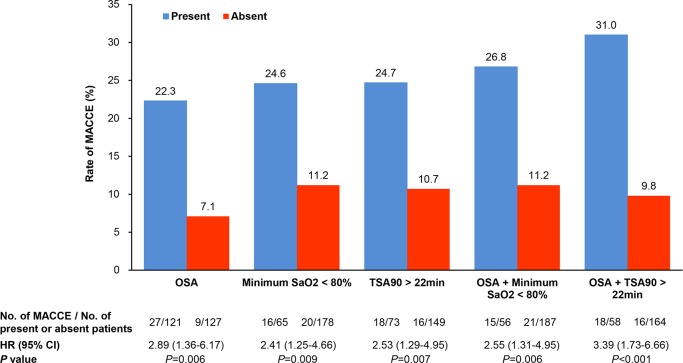
Rate of MACCE based on single or combined OSA characteristics in patients with diabetes. The best cut-off values of minimum SaO_2_ <80% and TSA90 >22 min were used in the adjusted Cox regression analysis. MACCE, major adverse cardiovascular and cerebrovascular event; OSA, obstructive sleep apnea; SaO_2_, arterial oxygen saturation; TSA90, the duration of time with SaO_2_ <90%.

ROC analysis was performed to identify OSA-related characteristics predictive of MACCE in patients with DM. The minimum SaO_2_ and the duration of time with SaO_2_ <90% (TSA90) that predicted MACCE was a cut-off value of 80% (AUC=0.603; p=0.048) and 22 min (AUC=0.639; p=0.01), respectively. The rate of MACCE based on single or combined OSA characteristics is shown in figure 3. The MACCE rate increased to 31.0% with concomitant OSA and TSA90 >22 min, compared with 9.8% when both were absent (HR=3.39, 95% CI 1.73 to 6.66, p<0.001).

## Discussion

The present analysis reveals a synergistic relationship between DM and OSA, such that patients with DM and OSA had a 2.5-fold higher rate of MACCE compared with patients with DM but without OSA after ACS onset. In contrast, among non-DM patients, the incidence of MACCE was similar between the OSA and non-OSA groups. The increased risk of adverse events was predominately isolated to OSA patients with poor glucose control. The presence of OSA combined with increased hypoxia duration further exacerbated this risk in patients with DM.

Several small observational studies have demonstrated the association between OSA and increased risk of adverse outcomes following ACS.^12–14^ Our study extends prior analysis by focusing on the role of OSA in a high-risk subset of patients with DM. The prevalence of OSA is high in patients with DM;^18 25 26^ both comorbidities accelerate progression of atherosclerosis,^2 10^ predisposing to recurrent events in those that develop ACS. In the present study, the presence of OSA significantly predicted subsequent cardiovascular events in patients with DM, but not in non-DM patients (consistent with the Sleep and Stent Study).^27^ Moreover, our study provided detailed information regarding glucose control and found that the increased risk of MACCE was observed only in patients with OSA with higher glucose or HbA1c levels. These results indicate that patients with combined OSA and DM represent a very high-risk group for future adverse events.

Emerging evidence has demonstrated a close relationship between OSA and incident diabetes.^28 29^ OSA-mediated intermittent hypoxemia and sleep fragmentation are likely contributing factors leading to abnormal glucose metabolism. Mechanistically, recurrent cycles of hypoxemia with reoxygenation promote oxidative stress, systemic inflammation, and endothelial dysfunction, all implicated in the pathogenesis of diabetes and related macrovascular and microvascular complications.^30^ Noteworthy, there is reverse causality between OSA and DM, as diabetes neuropathy may worsen OSA and nocturnal hypoxemia by affecting central control of respiration.^31–33^ The significant interaction between OSA and DM may exert a synergistic effect to promote the development and progression of atherosclerosis and increase ischemic events in patients with ACS. In our study, the presence of OSA and DM was associated with a very high rate of multivessel disease. Patients with concomitant OSA and DM were at the highest risk of incurring a MACCE. The combination of OSA and increased hypoxia duration increased the MACCE rate to 31.0% in patients with DM.

Specifically, most events in this study were rehospitalizations for unstable angina and repeat revascularization (mostly attributed to non-target vessels). Cardiovascular death, MI, and stroke were less common. Prior studies have demonstrated that long-term adverse outcomes in patients with DM were predominantly caused by new lesions in non-culprit vessels.^34^ Similarly, untreated OSA was also associated with increased risk of repeat revascularization (mainly in non-target lesions).^35^ Patients with OSA and DM, given shared mechanisms, are more prone to native plaque progression and subsequent cardiac ischemic events.

On the other hand, patients without DM represent a relatively low-risk subset compared with those with DM. According to intravascular ultrasound analysis, non-DM patients had fewer lesions across the coronary tree and had less necrotic core as well as a lower frequency of thin-cap fibroatheromas, indicating a lower risk of plaque vulnerability.^36 37^ Although OSA could promote oxidative stress and inflammatory responses, resulting in endothelial dysfunction and reduction of repair capacity,^38^ it may have less effect in non-DM patients. In our study, OSA was not associated with increased risk of cardiovascular events following ACS in non-DM patients, a finding also observed in another study including patients undergoing PCI.^27^ Thus, it is more reasonable and clinically significant to screen for OSA in high-risk patients with ACS (DM, etc).

Currently, data from randomized controlled trials do not support a role of CPAP for reducing cardiovascular events among patients with established cardiovascular disease.^39 40^ In the RICCADSA (Randomized Intervention with Continuous Positive Airway Pressure in CAD and OSA) Study, 224 non-sleepy patients with OSA and coronary artery disease (CAD) were randomized to CPAP or usual care. At a median follow-up of 57 months, no difference was found in a composite end point of repeat revascularization, MI, stroke, or cardiovascular death.^39^ The negative results may be partly explained by the fact that patients are under guideline-based optimal medical therapy, including intensive antiplatelet therapy, and control of blood pressure, dyslipidemia, and glucose, which are also the intermediate mechanisms implicated in OSA.^41^ More importantly, the RICCADSA trial included the entire spectrum of patients with CAD, with only 25% of patients having DM, thereby limiting the sample to a potentially lower-risk group.^39^ In the present study, only patients with combined DM and OSA had a significantly increased risk of future events after ACS, therefore representing a high-risk subset most likely to respond to the intervention. Conversely, patients with DM without OSA had a similar prognosis as patients without DM (with or without OSA). The presence of OSA seems to be a key modifier contributing to the negative effect of DM in patients with ACS. Thus, for patients with ACS and DM, it is important to screen for OSA and intervention may be needed, although more trials evaluating the effects of OSA treatment in this high-risk subgroup are warranted.

### Study limitations

First, the size of the DM cohort with 248 patients was modest. However, the primary outcome showed a significant difference between the OSA and non-OSA groups in patients with DM. Also, the results based on glucose and HbA1c levels were consistent with the main findings. Second, the diagnosis of OSA was based on portable polygraphy, a method that may underestimate AHI due to overestimating actual sleeping time. Third, although OSA severity may be overestimated during the acute setting of ACS but not stable CAD,^42–44^ this is true for OSA assessment in the setting of any high-risk acute disease including heart failure. Additionally, the patients in our study received a sleep study after clinical stabilization during hospitalization, minimizing the potential bias. Fourth, the overall sample was predominantly male and non-obese. Whether our findings may be extrapolated to the female or obese population is unknown. Finally, this study primarily recruited East-Asian patients. Studies pertaining to other ethnicities are needed.

## Conclusions

The present study demonstrated that in high-risk patients with ACS and DM, the presence of OSA was associated with a higher incidence of MACCE compared with patients without OSA. The increased risk associated with OSA was not observed in non-DM patients. Moreover, the prognosis of patients with DM, but without OSA, was similar to patients without DM. Further randomized trials exploring the efficacy of OSA treatment in the high-risk population with both DM and ACS are highly warranted.

## References

[1] Rana JS, Dunning A, Achenbach S, et al Differences in prevalence, extent, severity, and prognosis of coronary artery disease among patients with and without diabetes undergoing coronary computed tomography angiography: results from 10,110 individuals from the confirm (coronary CT angiography evaluation for clinical outcomes): an international multicenter registry. Diabetes Care 2012;35:1787–94. 10.2337/dc11-2403 22699296 PMC3402246

[2] Kim U, Leipsic JA, Sellers SL, et al Natural history of diabetic coronary atherosclerosis by quantitative measurement of serial coronary computed tomographic angiography: results of the paradigm study. JACC Cardiovasc Imaging 2018;11:1461–71. 10.1016/j.jcmg.2018.04.009 29778853

[3] Inaba S, Okayama H, Funada J-ichi, et al Impact of type 2 diabetes on serial changes in tissue characteristics of coronary plaques: an integrated Backscatter intravascular ultrasound analysis. Eur Heart J Cardiovasc Imaging 2012;13:717–23. 10.1093/ehjci/jes033 22368195

[4] Nicholls SJ, Tuzcu EM, Kalidindi S, et al Effect of diabetes on progression of coronary atherosclerosis and arterial remodeling: a pooled analysis of 5 intravascular ultrasound trials. J Am Coll Cardiol 2008;52:255–62. 10.1016/j.jacc.2008.03.051 18634979

[5] Jonas DE, Amick HR, Feltner C, et al Screening for obstructive sleep apnea in adults: evidence report and systematic review for the US preventive services Task force. JAMA 2017;317:415–33. 10.1001/jama.2016.19635 28118460

[6] Jensen LO, Maeng M, Thayssen P, et al Influence of diabetes mellitus on clinical outcomes following primary percutaneous coronary intervention in patients with ST-segment elevation myocardial infarction. Am J Cardiol 2012;109:629–35. 10.1016/j.amjcard.2011.10.018 22152969

[7] Kedhi E, Généreux P, Palmerini T, et al Impact of coronary lesion complexity on drug-eluting stent outcomes in patients with and without diabetes mellitus: analysis from 18 pooled randomized trials. J Am Coll Cardiol 2014;63:2111–8. 10.1016/j.jacc.2014.01.064 24632279

[8] Koo C-Y, de la Torre AS, Loo G, et al Effects of ethnicity on the prevalence of obstructive sleep apnoea in patients with acute coronary syndrome: a pooled analysis of the ISAACC trial and sleep and stent study. Heart Lung Circ 2017;26:486–94. 10.1016/j.hlc.2016.09.010 27939743

[9] Weinreich G, Wessendorf TE, Erdmann T, et al Association of obstructive sleep apnoea with subclinical coronary atherosclerosis. Atherosclerosis 2013;231:191–7. 10.1016/j.atherosclerosis.2013.09.011 24267225

[10] Tan A, Hau W, Ho H-H, et al OSA and coronary plaque characteristics. Chest 2014;145:322–30. 10.1378/chest.13-1163 24178625

[11] Arzt M, Hetzenecker A, Steiner S, et al Sleep-disordered breathing and coronary artery disease. Can J Cardiol 2015;31:909–17. 10.1016/j.cjca.2015.03.032 26112301

[12] Mazaki T, Kasai T, Yokoi H, et al Impact of sleep-disordered breathing on long-term outcomes in patients with acute coronary syndrome who have undergone primary percutaneous coronary intervention. J Am Heart Assoc 2016;5:e003270 10.1161/JAHA.116.003270 27307401 PMC4937269

[13] Lee C-H, Khoo S-M, Chan MY, et al Severe obstructive sleep apnea and outcomes following myocardial infarction. J Clin Sleep Med 2011;7:616–21. 10.5664/jcsm.1464 22171200 PMC3227707

[14] Yumino D, Tsurumi Y, Takagi A, et al Impact of obstructive sleep apnea on clinical and angiographic outcomes following percutaneous coronary intervention in patients with acute coronary syndrome. Am J Cardiol 2007;99:26–30. 10.1016/j.amjcard.2006.07.055 17196456

[15] Wang X, Fan J-Y, Zhang Y, et al Association of obstructive sleep apnea with cardiovascular outcomes after percutaneous coronary intervention: a systematic review and meta-analysis. Medicine 2018;97:e0621 10.1097/MD.0000000000010621 29703065 PMC5944507

[16] Lee C-H, Sethi R, Li R, et al Obstructive sleep apnea and cardiovascular events after percutaneous coronary intervention. Circulation 2016;133:2008–17. 10.1161/CIRCULATIONAHA.115.019392 27178625

[17] Tasali E, Mokhlesi B, Van Cauter E Obstructive sleep apnea and type 2 diabetes: interacting epidemics. Chest 2008;133:496–506. 10.1378/chest.07-0828 18252916

[18] Grimaldi D, Beccuti G, Touma C, et al Association of obstructive sleep apnea in rapid eye movement sleep with reduced glycemic control in type 2 diabetes: therapeutic implications. Diabetes Care 2014;37:355–63. 10.2337/dc13-0933 24101701 PMC3898763

[19] Altaf QA, Dodson P, Ali A, et al Obstructive sleep apnea and retinopathy in patients with type 2 diabetes. A longitudinal study. Am J Respir Crit Care Med 2017;196:892–900. 10.1164/rccm.201701-0175OC 28594570 PMC5649977

[20] Tahrani AA, Ali A, Raymond NT, et al Obstructive sleep apnea and diabetic neuropathy: a novel association in patients with type 2 diabetes. Am J Respir Crit Care Med 2012;186:434–41. 10.1164/rccm.201112-2135OC 22723291 PMC3443800

[21] Fan J, Wang X, Ma X, et al Association of obstructive sleep apnea with cardiovascular outcomes in patients with acute coronary syndrome. J Am Heart Assoc 2019;8:e010826 10.1161/JAHA.118.010826 30636505 PMC6497330

[22] Ibanez B, James S, Agewall S, et al 2017 ESC guidelines for the management of acute myocardial infarction in patients presenting with ST-segment elevation: the task force for the management of acute myocardial infarction in patients presenting with ST-segment elevation of the European Society of cardiology (ESC). Eur Heart J 2018;39:119–77. 10.1093/eurheartj/ehx393 28886621

[23] Roffi M, Patrono C, Collet J-P, et al 2015 ESC guidelines for the management of acute coronary syndromes in patients presenting without persistent ST-segment elevation: Task force for the management of acute coronary syndromes in patients presenting without persistent ST-segment elevation of the European Society of cardiology (ESC). Eur Heart J 2016;37:267–315. 10.1093/eurheartj/ehv320 26320110

[24] Hicks KA, Tcheng JE, Bozkurt B, et al 2014 ACC/AHA key data elements and definitions for cardiovascular endpoint events in clinical trials: a report of the American College of Cardiology/American heart association Task force on clinical data standards (writing Committee to develop cardiovascular endpoints data standards). J Am Coll Cardiol 2015;66:403–69. 10.1016/j.jacc.2014.12.018 25553722

[25] Zhang P, Zhang R, Zhao F, et al The prevalence and characteristics of obstructive sleep apnea in hospitalized patients with type 2 diabetes in China. J Sleep Res 2016;25:39–46. 10.1111/jsr.12334 26268508

[26] Lam DCL, Lui MMS, Lam JCM, et al Prevalence and recognition of obstructive sleep apnea in Chinese patients with type 2 diabetes mellitus. Chest 2010;138:1101–7. 10.1378/chest.10-0596 20705796

[27] Koo CY, Drager LF, Sethi R, et al Obstructive sleep apnea and diabetes independently add to cardiovascular risk after coronary revascularization. Diabetes Care 2018;41:e12–14. 10.2337/dc17-0759 29208655

[28] Kendzerska T, Gershon AS, Hawker G, et al Obstructive sleep apnea and incident diabetes. A historical cohort study. Am J Respir Crit Care Med 2014;190:218–25. 10.1164/rccm.201312-2209OC 24897551

[29] Nagayoshi M, Punjabi NM, Selvin E, et al Obstructive sleep apnea and incident type 2 diabetes. Sleep Med 2016;25:156–61. 10.1016/j.sleep.2016.05.009 27810258 PMC5102826

[30] Reutrakul S, Mokhlesi B Obstructive sleep apnea and diabetes: a state of the art review. Chest 2017;152:1070–86. 10.1016/j.chest.2017.05.009 28527878 PMC5812754

[31] Bottini P, Redolfi S, Dottorini ML, et al Autonomic neuropathy increases the risk of obstructive sleep apnea in obese diabetics. Respiration 2008;75:265–71. 10.1159/000100556 17347559

[32] Resnick HE, Redline S, Shahar E, et al Diabetes and sleep disturbances: findings from the sleep heart health study. Diabetes Care 2003;26:702–9. 10.2337/diacare.26.3.702 12610025

[33] Bottini P, Dottorini ML, Cristina Cordoni M, et al Sleep-disordered breathing in nonobese diabetic subjects with autonomic neuropathy. Eur Respir J 2003;22:654–60. 10.1183/09031936.03.00070402 14582920

[34] Kedhi E, Kennedy MW, Maehara A, et al Impact of TCFA on Unanticipated ischemic events in medically treated diabetes mellitus: insights from the PROSPECT study. JACC Cardiovasc Imaging 2017;10:451–8. 10.1016/j.jcmg.2015.12.023 27372016

[35] Wu X, Lv S, Yu X, et al Treatment of OSA reduces the risk of repeat revascularization after percutaneous coronary intervention. Chest 2015;147:708–18. 10.1378/chest.14-1634 25412159 PMC4347533

[36] Marso SP, Mercado N, Maehara A, et al Plaque composition and clinical outcomes in acute coronary syndrome patients with metabolic syndrome or diabetes. JACC Cardiovasc Imaging 2012;5:S42–52. 10.1016/j.jcmg.2012.01.008 22421230

[37] Nasu K, Tsuchikane E, Katoh O, et al Plaque characterisation by virtual histology intravascular ultrasound analysis in patients with type 2 diabetes. Heart 2008;94:429–33. 10.1136/hrt.2007.118950 17646194

[38] Kohler M, Stradling JR Mechanisms of vascular damage in obstructive sleep apnea. Nat Rev Cardiol 2010;7:677–85. 10.1038/nrcardio.2010.145 21079639

[39] Peker Y, Glantz H, Eulenburg C, et al Effect of positive airway pressure on cardiovascular outcomes in coronary artery disease patients with Nonsleepy obstructive sleep apnea. The RICCADSA randomized controlled trial. Am J Respir Crit Care Med 2016;194:613–20. 10.1164/rccm.201601-0088OC 26914592

[40] McEvoy RD, Antic NA, Heeley E, et al Cpap for prevention of cardiovascular events in obstructive sleep apnea. N Engl J Med 2016;375:919–31. 10.1056/NEJMoa1606599 27571048

[41] Drager LF, McEvoy RD, Barbe F, et al Sleep apnea and cardiovascular disease: lessons from recent trials and need for team science. Circulation 2017;136:1840–50. 10.1161/CIRCULATIONAHA.117.029400 29109195 PMC5689452

[42] Low T-T, Hong W-Z, Tai B-C, et al The influence of timing of polysomnography on diagnosis of obstructive sleep apnea in patients presenting with acute myocardial infarction and stable coronary artery disease. Sleep Med 2013;14:985–90. 10.1016/j.sleep.2013.03.025 23890600

[43] Schiza SE, Simantirakis E, Bouloukaki I, et al Sleep disordered breathing in patients with acute coronary syndromes. J Clin Sleep Med 2012;8:21–6. 10.5664/jcsm.1652 22334805 PMC3266342

[44] Buchner S, Greimel T, Hetzenecker A, et al Natural course of sleep-disordered breathing after acute myocardial infarction. Eur Respir J 2012;40:1173–9. 10.1183/09031936.00172211 22441744

