# Supplementary material for: Clinical significance of obstructive sleep apnea in patients with acute coronary syndrome in relation to diabetes status
Source: BMJ Open Diabetes Res Care. 2019 Dec 18;7(1):e000737. doi: 10.1136/bmjdrc-2019-000737 (PMC6936388; doi:10.1136/bmjdrc-2019-000737)
Supplement: Supplementary data [file bmjdrc-2019-000737supp001.pdf]

eTable 1. Baseline Demographic, Clinical, and Procedural Characteristics by Diabetic Status

Variables	All (N=804)	Diabetes (n=248)	No Diabetes (n=556)	P value
**Demographics**				
Age, mean±SD, years	57.5±10.2	59.1±9.7	56.7±10.4	0.003
Male	664 (82.6)	188 (75.8)	476 (85.6)	0.001
Height, mean±SD, cm	168.5±7.4	167.7±7.6	168.8±7.3	0.08
Weight, mean±SD, kg	76.0±12.2	75.5±11.1	76.2±12.7	0.49
BMI, mean±SD, kg/m^2	26.7±3.6	26.8±3.1	26.7±3.7	0.80
Waist-to-hip ratio, median (IQR)	0.98 (0.96-1.02)	0.99 (0.95-1.02)	0.98 (0.95-1.02)	0.34
Neck circumference, median (IQR), cm	40 (38-42)	40 (38-42)	40 (37-41)	0.60
Systolic BP, median (IQR), mmHg	125 (115-138)	128 (116-140)	125 (115-138)	0.16
Diastolic BP, median (IQR), mmHg	74 (70-84)	73 (67-83)	74 (70-85)	0.13
**Medical history**				
Hypertension	530 (65.9)	187 (75.4)	343 (61.7)	<0.001
Hyperlipidemia	210 (26.1)	84 (33.9)	126 (22.7)	0.001
Family history of premature CAD	59 (7.3)	15 (6.0)	44 (7.9)	0.35
Prior stroke	76 (9.5)	37 (14.9)	39 (7.0)	<0.001
Prior myocardial infarction	118 (14.7)	42 (16.9)	76 (13.7)	0.23
Prior PCI	141 (17.5)	51 (20.6)	90 (16.2)	0.13

Prior CABG	11 (1.4)	8 (3.2)	3 (0.5)	0.005
Smoking				0.03
No	288 (35.8)	105 (42.3)	183 (32.9)	
Current	406 (50.5)	110 (44.4)	296 (53.2)	
Previous	110 (13.7)	33 (13.3)	77 (13.8)	
**Baseline lab tests**				
Glucose, median (IQR), mmol/L	5.9 (5.3-7.3)	8.0 (6.3-10.5)	5.6 (5.1-6.2)	<0.001
Hemoglobin A1c, median (IQR), %	6.0 (5.6-6.9)	7.5 (6.6-8.9)	5.8 (5.5-6.1)	<0.001
Hs-CRP, median (IQR), mg/L	2.3 (0.8-8.6)	2.0 (0.8-6.5)	2.5 (0.8-9.7)	0.37
**Echocardiographic findings**				
LVEF, median (IQR), %	60 (55-65)	60 (55-66)	60 (55-65)	0.27
LVEDD, median (IQR), mm	49 (46-52)	48 (45-51)	49 (46-52)	0.02
**Diagnosis**				0.02
Unstable angina	347 (43.2)	125 (50.4)	222 (39.9)	
NSTEMI	203 (25.2)	59 (23.8)	144 (25.9)	
STEMI	254 (31.6)	64 (25.8)	190 (34.2)	
**Procedures**				
Coronary angiography	786 (97.8)	242 (97.6)	544 (97.8)	0.82
Multivessel disease	61.2 (481/786)	157/242 (64.9)	324/544 (59.6)	0.16

PCI	490 (60.9)	140 (56.5)	351 (63.1)	0.07
Stenting	432 (53.7)	118 (47.6)	314 (56.5)	0.02
Stents implanted, median (IQR), n	1.0 (1.0-2.0)	1.0 (1.0-2.0)	1.0 (1.0-2.0)	>0.99
CABG	80 (10.0)	31 (12.5)	49 (8.8)	0.11
**Sleep study**				
OSA (AHI≥15)	403 (50.1)	121 (48.8)	282 (50.7)	0.61
AHI, median (IQR), events·h^{-1}	15.0 (7.4-31.2)	14.4 (6.7-31.4)	15.2 (7.7-31.2)	0.52
ODI, median (IQR), events·h^{-1}	14.4 (7.4-29.5)	12.9 (6.2-28.5)	15.4 (8.0-29.7)	0.20
Minimum SaO_2, median (IQR), %	85 (79-88)	85 (79-88)	85 (80-88)	0.96
Mean SaO_2, median (IQR), %	94 (93-95)	94 (93-95)	94 (93-95)	0.81
Time with SaO_2<90%, median (IQR), minute	5.5 (0.5-31.9)	5.9 (0.5-35.5)	5.5 (0.6-30.3)	0.95
Epworth Sleepiness Scale, mean±SD	8.3±5.0	8.2±5.0	8.3±5.1	0.88
**Medications on discharge**				
Aspirin	754 (93.8)	228 (91.9)	526 (94.6)	0.15
Thienopyridine	720 (89.6)	218 (87.9)	502 (90.3)	0.31
β-Blockers	611 (76.0)	188 (75.8)	423 (76.1)	0.93
ACEIs/ARBs	564 (70.1)	179 (72.2)	385 (69.2)	0.40
Statins	762 (94.8)	230 (92.7)	532 (95.7)	0.08

Data are presented as mean±SD, median (IQR), n (%), or n/N (%). ACEI indicates angiotensin-converting enzymes inhibitor; AHI, apnea-hypopnea index; ARB, angiotensin receptor blocker; BMI, body mass index; BP, blood pressure; CABG, coronary artery bypass grafting; CAD, coronary artery disease; Hs-CRP, high-sensitivity C-reactive protein; IQR, interquartile range; LVEDD, left ventricular end-diastolic dimension; LVEF, left ventricular ejection fraction; NSTEMI, non-ST-segment elevation myocardial infarction; ODI, oxygen desaturation index; OSA, obstructive sleep apnea; PCI, percutaneous coronary intervention; SaO2, arterial oxygen saturation; SD, standard deviation; STEMI, ST-segment-elevation myocardial infarction.

eTable 2. Results of Sleep Study in OSA Versus Non-OSA Groups According to Diabetes Status

Variables	All (N=804)	Diabetes (n=248)			No Diabetes (n=556)		
		OSA (n=121)	Non-OSA (n=127)	P value	OSA (n=282)	Non-OSA (n=274)	P value
AHI, median (IQR), events·h^{-1}	15.0 (7.4-31.2)	31.9 (22.3-45.8)	6.9 (3.6-9.8)	<0.001	30.2 (21.7-42.8)	7.6 (3.9-10.8)	<0.001
ODI, median (IQR), events·h^{-1}	14.4 (7.4-29.5)	30.4 (20.5-46.7)	6.8 (4.0-10.3)	<0.001	29.4 (21.6-41.9)	8.0 (4.2-11.5)	<0.001
Minimum SaO_2, median (IQR), %	85 (79-88)	80 (73-85)	88 (85-89)	<0.001	82 (76-86)	87 (84-90)	<0.001
Mean SaO_2, median (IQR), %	94 (93-95)	93 (92-94)	95 (94-96)	<0.001	93 (92-94)	95 (94-96)	<0.001
Time with SaO_2<90%, median (IQR), minute	5.5 (0.5-31.9)	28.4 (8.8-66.1)	1.0 (0.0-5.7)	<0.001	23.9 (5.2-63.2)	1.2 (0.0-6.7)	<0.001
Epworth Sleepiness Scale, mean±SD	8.3±5.0	8.8±5.1	7.7±4.8	0.27	8.9±4.9	7.7±5.2	0.044

Data are presented as mean±SD or median (IQR). AHI indicates apnea-hypopnea index; IQR, interquartile range; ODI, oxygen desaturation index; OSA, obstructive sleep apnea; SaO_2, arterial oxygen saturation; SD, standard deviation.

eTable 3. Crude Number of Events in OSA Versus Non-OSA Groups by Diabetes Status

Variables	All (N=804)	Diabetes (n=248)		No Diabetes (n=556)	
		OSA (n=121)	Non-OSA (n=127)	OSA (n=282)	Non-OSA (n=274)
MACCE	81 (10.1)	27 (22.3)	9 (7.1)	24 (8.5)	21 (7.7)
Cardiovascular death	11 (1.4)	5 (4.1)	1 (0.8)	0 (0.0)	5 (1.8)
Myocardial infarction	11 (1.4)	3 (2.5)	1 (0.8)	1 (0.4)	6 (2.2)
Stroke	9 (1.1)	4 (3.3)	1 (0.8)	2 (0.7)	2 (0.7)
Ischemic	6 (1.0)	3 (2.5)	1 (0.8)	1 (0.4)	1 (0.4)
Hemorrhagic	3 (0.4)	1 (0.8)	0 (0.0)	1 (0.4)	1 (0.4)
Hospitalization for unstable angina	49 (6.1)	16 (13.2)	6 (4.7)	17 (6.0)	10 (3.6)
Hospitalization for heart failure	6 (0.7)	1 (0.8)	0 (0.0)	2 (0.7)	3 (1.1)
Ischemia-driven revascularization	27 (3.4)	8 (6.6)	5 (3.9)	9 (3.2)	5 (1.8)
All-cause mortality	11 (1.4)	5 (4.1)	1 (0.8)	0 (0.0)	5 (1.8)
All repeat revascularization	48 (6.0)	11 (9.1)	6 (4.7)	17 (6.0)	14 (5.1)
Target vessel revascularization	14 (1.7)	4 (3.3)	3 (2.4)	5 (1.8)	2 (0.7)
Non-target vessel revascularization	40 (5.0)	9 (7.4)	3 (2.4)	14 (5.0)	14 (5.1)
PCI	42 (5.2)	11 (9.1)	6 (4.7)	15 (5.3)	10 (3.6)
CABG	6 (0.7)	0 (0.0)	0 (0.0)	2 (0.7)	4 (1.5)

Composite of all events	100 (12.4)	29 (24.0)	10 (7.9)	32 (11.3)	29 (10.6)

Data are presented as n (%). MACCE indicates major adverse cardiovascular and cerebrovascular event; CABG, coronary artery bypass grafting; PCI, percutaneous coronary intervention; OSA, obstructive sleep apnea.
